# Parenting Sense of Competence: Psychometrics and Invariance among a Community and an At-Risk Samples of Portuguese Parents

**DOI:** 10.3390/healthcare11010015

**Published:** 2022-12-21

**Authors:** Cristina Nunes, Lara Ayala-Nunes, Laura Inês Ferreira, Pedro Pechorro, Délia Freitas, Cátia Martins, Rita Santos

**Affiliations:** 1 Psychology Research Centre (CIP), Universidade do Algarve, 8005-135 Faro, Portugal; 2 CINEICC, PsyAssessmentLab., Universidade de Coimbra, 3000-115 Coimbra, Portugal; 3 Departamento de Psicologia e Ciências da Educação, Universidade do Algarve, 8005-135 Faro, Portugal

**Keywords:** at-risk families, efficacy, instrumental study, parenting competences, PSOC, satisfaction

## Abstract

Parenting sense of competence refers to parents’ perception about their ability to perform the parenting role, one of the key dimensions in family dynamics. This construct is even more important in families at psychosocial risk, where the exercise of parenting can be more challenging. The Parenting Sense of Competence scale (PSOC) is a self-report measure that aims to assesses one’s perceived efficacy and satisfaction with parenting. In this cross-sectional and instrumental study, we aimed to examine the psychometric properties of the PSOC among a Portuguese sample of community (*n* = 205) and at-psychological-risk (*n* = 273) parents. Participants completed the PSOC, the Enrich Marital Satisfaction Scale, the Parenting Stress Index, and the Parenting Alliance Inventory. Results from confirmatory factor analysis showed that a two-factor revised model obtained the best fit, with some items being removed. Our data confirmed that the PSOC has good psychometric properties, with acceptable reliability and validity and measurement invariance across the community and the at-risk samples. Findings of this psychometric exploration provided evidence that the PSOC is a reliable measure of easy application and interpretation for assessing the perceived competence of Portuguese parents.

## 1. Introduction

Parenting sense of competence (PSC) is a cognitive and emotional construct that refers to the judgments that parents hold about their abilities as caregivers. It also includes parents’ beliefs about their capacity to positively influence their children’s development and their satisfaction with the parenting role [1,2,3].

This construct has been widely studied and is a relevant dimension for the assessment and understanding of family dynamics. Parenting sense of competence has been associated to several family dimensions, such as marital relationship and family functioning [2,4,5]. For instance, mothers’ sense of competence has been positively linked to coparenting support [6], and a reciprocal relationship between perceived parental competence and marital stress over a 6-year interval has been reported for both mothers and fathers [7].

It is especially important to assess this construct in families who are at psychosocial risk [8,9,10,11,12]—i.e., families that have difficulties in adequately meeting children’s needs but not severely enough to require children’s placement in foster care [13]. This is because research has suggested indirect relationships between PSC and potential for child abuse [14] and maltreatment [15]. Similarly, PSC is thought to be a protective factor for negative outcomes, buffering the impact of risk factors such as maternal depression, children’s difficult temperament, and disadvantaged environments [16,17].

It has been associated with a range of parental adjustment dimensions, such as mental health, e.g., depression [18,19], and parenting stress [20,21,22]. An inverse correlation between PSC and depression has also been repeatedly observed [23,24] as well as moderate prospective effect sizes between these two variables [25]. PSC has also been found to mediate the effects of maternal depression on mothers’ parenting competence [24]. Complex links between parenting stress and PSC have been proposed as a predictor, mediator, and consequence of parenting stress [21]. PSC has also been shown to influence a wide range of parenting behaviors and skills [26,27,28,29,30] at different developmental stages. PSC has been shown to be positively associated to parental warmth and control with toddlers [31], to positive parenting of pre-school children [32], and to parental involvement and monitoring of adolescents [30]. Conversely, PSC has been found to be negatively correlated with negative parenting styles in mothers with severe mental illnesses [29].

There are consequences to children’s development and well-being that are induced by PSC. Specifically, some studies have found a positive relationship between PSC and children’s adaptive behaviors [26,33], greater learning achievements and social and emotional skills [16,34], well-being [18,35,36], and physical health [37].

According to Ardelt and Eccles’ [16] conceptual model, parents who feel competent are more likely to engage in promotive parenting strategies—i.e., strategies that foster social competence and cultivate children’s skills and talents—which will favorably impact their children’s socio-emotional and academic adjustment. A high PSC can also impact children directly through modelling of attitudes and beliefs about their own agency and self-efficacy. In contrast, a perception of low PSC may lead these parents to become less involved in positive parenting practices or feel more unmotivated and discouraged when facing difficulties in exercising their parenting role.

One of the most used scales to measure this construct is Johnston and Mash’s [38] version of the Parenting Sense of Competence (PSOC) questionnaire. This scale consists of 16 items and evaluates the caregiver’s perceived parenting competence through its two dimensions: efficacy, i.e., the degree to which the parent feels competent in their parenting role (7 items, e.g., “The problems of taking care of a child are easy to solve once you know how your actions affect your child, an understanding I have acquired.”), and satisfaction, i.e., the extent to which the caregiver feels satisfied with their role as a parent (9 items, e.g., “Even though being a parent could be rewarding, I am frustrated now while my child is at his/her present age.”).

This scale has been validated in several countries, such as Australia [39,40], Brazil [41,42], Canada [4,38], China [43], Spain [44,45,46], Thailand [47], and Uganda [48]. There have been several adaptations of this scale in Portugal, with community samples [49,50] and families at psychosocial risk [51]. Ferreira and colleagues [49] conducted a confirmatory factor analysis and proposed a three-factor solution of satisfaction (α = 0.84), efficacy (α = 0.87), and interest (α = 0.82), including only 15 out of the 16 original items (item 8 removed). Seabra-Santos and colleagues [50] aimed at reinforcing the validation and accuracy studies of the PSOC and, after conducting a confirmatory factor analysis, found support for a two-factor solution of satisfaction (α = 0.75) and efficacy (α = 0.72), including all 16 items. A study that aimed at adapting for the PSOC to families at psychosocial risk by Nunes and colleagues [51] performed an exploratory factor analysis and proposed a three-factor solution including only 14 items: efficacy (α = 0.74), dissatisfaction (α = 0.72), and controllability of the child-rearing task (α = 0.65). In Nunes and colleagues’ [51] version, items 8 and 16 were removed due to low factor loading. Problems with these items were found in previous studies [4,40,49].

Although there are several Portuguese adaptations and validation studies of the PSOC, the factorial solutions and proposed dimensions are not consensual. Furthermore, these studies had limitations that should be overcome: the validations conducted with the general population only included parents of pre-school aged children [49,50], and the adaptation with families at psychosocial risk had a sample comprised exclusively of mothers [51]. Having a Portuguese version of the PSOC validated with a broader children’s age range and with both mothers and fathers would allow to reliably measure the change in PSC as an outcome of parenting programs, thus providing researchers and practitioners with an additional tool to evaluate the efficacy of family interventions.

This study intended to explore the two-factor structure of PSOC using a Portuguese sample of parents from the community and at-risk families while also examining the measurement invariance. Using a confirmatory factorial analysis, it was hypothesized that the PSOC would show: (1) a two-factor solution for both community and at-risk parents; (2) the supposed measurement invariance among samples; (3) satisfactory internal consistency; (4) criteria validity (e.g., positive associations with measures of marital satisfaction and parenting alliance and a negative association with a parenting stress index); and (5) different scores of participants from the community and the at-risk sample in the satisfaction subscale of the PSOC, with parents from the community sample showing higher satisfaction than at-risk parents. 

## 2. Materials and Methods

The present study is cross-sectional and descriptive, with a simple retrospective investigation plan. It is also an instrumental study because it aims to assess the psychometric characteristics of a measure [52].

### 2.1. Participants

A total of 205 parents from the community (Mage = 38.39 years; SD = 5.84 years; age range = 23–53 years) agreed to participate in this study. Just over half were women (52.20%) and had a medium-high education level: 42% completed secondary education, and 28.30% completed higher education studies. Their children had a mean age of 9.65 years (SD = 4.99; range = 1–18), and 52.68% were boys. An at-risk sample was also included, composed by 273 parents of Child Welfare Services (CWS)-referred children (Mage = 37.05 years; SD = 8.07 years; age range = 19–58 years). Participants were mostly women (80.05%), with a low educational level: 52% had not completed primary education, and 30.60% had completed primary education only. Their children had a mean age of 10.83 years (SD = 4.68; age range = 1–18), and more than half were boys (58.82%). At-risk parents refer to those who face serious problems and accumulate multiple stressful life events (e.g., marital conflict, abuse, economic problems, violent neighborhood, or inadequate social networks). These personal and relational circumstances in which they live hinder or limit their parenting skills; however, the situation is not severe enough to require child out-of-home placement [53].

There were no significant differences between the community and the at-risk samples regarding parents’ age (F = 3.99; *p* = n.s.), children’s age (F = 0.99; *p* = n.s.), or child’s gender (χ^2^(1, N = 477) = 1.79, *p* = n.s.). Significant differences were only found regarding parents’ years of education (χ^2^(3, N = 476) = 153.11, *p* = 0.000) and gender distribution (χ^2^(1, N = 476) = 49.01, *p* = 0.000).

### 2.2. Measures

#### 2.2.1. Parenting Sense of Competence (PSOC; Johnston and Mash [38]; Portuguese Version: Nunes and Colleagues [51])

As described before, the 16-item version of the PSOC scale of Johnston and Mash [38] was used, measuring efficacy (7 items, e.g., “I honestly believe I have all the skills necessary to be a good parent to my child”) and satisfaction (9 items, e.g., “My talents and interests are in other areas, not in being a parent”) with parenting. For both subscales, items are rated on a 6-point Likert scale from “1 = Strongly disagree” to “6 = Strongly agree”. Scores of satisfaction scale need to be reversed, and a total score can be computed. Higher scores indicate a higher sense of parenting competence.

The Portuguese version was developed by a process of forward-backward translation previously described elsewhere [51]. The present study showed acceptable values of internal consistency, with α = 0.74 for efficacy and α = 0.84 for satisfaction for the community sample and α = 0.74 for efficacy and α = 0.70 for satisfaction for the at-risk sample (the 16 items included).

#### 2.2.2. Parenting Alliance Inventory (PAI; Abidin and Brunner [54] Portuguese Version: Nunes, Ayala-Nunes and Colleagues [55])

The PAI is a self-report measure comprising 20 items that assess the degree of commitment and cooperation between both parents in childrearing (e.g., “When there is a problem with our child, we work out a good solution together”). Each item is rated on a 5-point scale, in which 1 means “Strongly agree”, and 5 means “Strongly disagree”. A total score is obtained by adding the 20 items, in which higher scores indicate stronger support between partners as parents. Internal consistency for the present study, estimated by Cronbach’s alpha, was α = 0.96 for the at-risk sample and α = 0.95 for the community sample.

#### 2.2.3. Enrich Marital Satisfaction Scale (EMS; Fowers and Olson [56]; Portuguese Version: Nunes and Colleagues [57])

The EMS is a measure consisting of 15 items that assesses the global satisfaction with the marital relationship, divided into the subscales marital satisfaction (MS, 10 items, e.g., “I am very satisfied with our way of making decisions and solving problems”) and idealized distortion (ID, 5 items, e.g., “Our relationship is perfect”). Items are quoted on a scale ranging from “1 = No, totally disagree” to “5 = Yes, totally agree”. Higher scores indicate higher marital satisfaction. In this study, the total score of the EMS was used, obtaining the following reliability indices: for the community sample, α = 0.89, and for the at-risk sample, α = 0.93.

#### 2.2.4. Parenting Stress Index—Short Form (PSI-SF; Abidin [58], Portuguese Version: Santos [59])

The PSI-SF is a self-report measure for the evaluation of three dimensions of stress associated with the parenting role: parental distress (e.g., “I feel trapped by my responsibilities as a parent”), parent–child dysfunctional interaction (e.g., “Sometimes I feel my child doesn’t like me and doesn’t want to be close to me”), and perception of the child as a difficult child (e.g., “My child makes more demands on me than most children”). It is composed by 36 items, scored in a 5-point scale (from “1 = Strongly disagree” to “5 = Strongly agree”), in which higher scores suggest greater levels of stress experienced from parenting. In the present study, the internal consistency for the total PSI-SF was α = 0.91 for the community sample and α = 0.90 for the at-risk sample.

A sociodemographic questionnaire was also applied to gather information about parents’ and children’s gender, age, and parents’ years of schooling and socioeconomic status.

### 2.3. Procedures

This research was approved by the Scientific Council of Psychology and Educational Sciences Department of the University of Algarve (Ref. No. 55_20/12/2017). Using a snowball sampling technique, master’s students from the Psychology Department of the University of Algarve were contacted and invited to participate in this study by recruiting five parents from the community living in the Algarve (South of Portugal). Each student was asked to recruit five parents.

For the at-risk sample, mothers and fathers with at least one child referred to CWS from Algarve were contacted and invited to participate in the study by their case manager from CWS from Algarve (South of Portugal). For parents to be included in this sample, they had to meet the following criteria: (1) belong to the CWS for follow-up due to family preservation for at least three months prior to data collection and (2) not be in a time of family crisis at the time of invitation and participation in the study (i.e., any significant changes in family situation that would increase the likelihood of a child out of the home).

Participants were informed about the aims of the study, its non-compensatory nature, the anonymous and confidential nature of their responses, as well as the possibility of withdrawing from the study at any time without any consequences. Data collection took place between January 2018 and February 2019. 

### 2.4. Data Analysis

Data analyses were performed with using IBM SPSS 24.0 (IBM Corp., Chicago, IL, USA) and EQS 6.3 [60]. The statistical assumptions for the parametric analyses were ascertained following the recommendations of Tabachnick and Fidell [52], with satisfactory results. Regarding missing values, we checked that data were missing completely at random using Little’s MCAR test (*p* = 0.08). After that, data on the item level were extrapolated using the EM algorithm for missing value analysis of SPSS. However, in cases where more than 10% of the items were missing, they were excluded from the analyses, resulting in two deleted cases. 

To analyze the factor structure of the PSOC Portuguese version, a confirmatory factor analysis (CFA) with ML robust estimation methods was conducted, using a categorical correlation matrix [38,51]. Goodness of fit indices were computed, including comparative fit index (CFI), Satorra–Bentler chi-square/degrees of freedom, incremental fit index (IFI), Akaike information criterion (AIC), and root mean square error of approximation (RMSEA). Regarding the IFI, also known as Bollen’s IFI, values that exceed 0.90 were regarded as acceptable. Regarding the Akaike information criterion (AIC), which aims to measure the discrepancy between the true model and the hypothesized model, the model with the smallest AIC should be selected [61]. A criterion of factor loadings above 0.30 was taken to retain items. Modification indexes (MI) were considered to examining possible changes to improve the measurement model [62].

To test the measurement invariance across the at-risk and the community samples [63], the ΔCFI and the ΔRMSEA were used considering the following criteria: ΔCFI < 0.01, ΔRMSEA < 0.015, RMSEA < 0.08, and CFI > 0.90. Further, the S-Bχ^2^ difference test was used to investigate if the constraints significantly weakened the model. 

Pearson correlations were used to analyze the associations between variables. Univariate analysis of variance tests were used to compare groups. Effect size calculations (i.e., η^2^ and r) were performed to clarify the degree of accuracy of the statistical judgments and the strength of the relationship between the variables.

## 3. Results

First, the psychometric properties of the PSOC were examined with CFA to replicate the different factor structures that have been proposed for this scale. Table 1 presents the four tested models, showing that the model with the best fit corresponds to the revised two-factor model, consisting of 10 items that already composed the original two-factor PSOC.

From the original structure, items that presented low loadings and low corrected item-total correlations were excluded (items 1, 2, 6, 8, 12, 16; see note on Table 2), and an intercorrelation error between items 13 and 14 was performed to improve the measurement model. 

Standardized item loadings for the two-factor model structure estimated with the ML Robust method are displayed in Table 2. All items had loadings well ≥0.30, and thus, none were removed from the model.

Second, we tested for measurement invariance across samples (at-risk versus community) using the revised two-factor model. 

The baseline model for comparisons was the configural model, with no constrains included. Fit indices of the configural model were compared with stricter models of weak or metric invariance (in which factor loadings were equally constrained among groups) and strong or scalar invariance (where both factor loadings and covariances were forced to be equal in the two groups).

Measurement invariance in both at-risk and community samples was confirmed through the values obtained in the Cheung and Rensvold’s criteria [64], with ΔCFI not exceeding 0.01, ΔRMSEA less than 0.015, CFI above 0.90, and RMSEA below 0.08 (see Table 3). Moreover, this was reinforced by the non-significant values of ΔS-Bχ^2^(df) in case of weak and strong invariance.

The revised two-factor PSOC revealed an acceptable internal consistency in the two dimensions since the scores were ≥0.74 in the community sample and ≥0.70 in the at-risk sample (see Table 4). Average variance extracted is under 0.5, but composite reliability is higher than 0.6, so convergent validity of the construct can be considered adequate. Moreover, the mean inter-item correlations and corrected item–total correlation range showed satisfactory values considering Nunnally and Bernstein’s [65] recommendations. In both samples, mean inter-item correlations values were between 0.15 and 0.50, and corrected item–total correlations were above 0.20.

Pearson correlations between the efficacy and satisfaction subscales were significant and positive, with a low magnitude in both samples (at-risk *r* = 0.20, *p* = 0.001; community sample *r* = 0.12; *p* = 0.040).

The PSOC showed the expected significant associations with other parenting and family measures (see Table 5). Correlations between efficacy and satisfaction with measures of parenting alliance and marital satisfaction were significant and positive, with a small-to-moderate magnitude. In the at-risk sample, there were moderate significant correlations between the two dimensions of the PSOC and the Parenting Stress Index, whereas the community sample showed a non-significant negative correlation between the PSOC and the Parenting Stress Index.

Analyzing the comparisons of efficacy and satisfaction scores between the at-risk sample and the community sample (Table 6), it was observed that community parents had a significantly higher score in parental role satisfaction (*M* = 21.63, *SD* = 4.58) compared with parents from at-risk families (*M* = 18.35, *SD* = 5.12, *F* = 52.62, *p* = 0.000, *η*^2^ = 0.10), with a moderate size effect. No significant differences were found on parenting efficacy.

Comparisons of mothers’ and fathers’ parenting efficacy and satisfaction showed no significant differences in either sample (Table 7).

## 4. Discussion

Parenting sense of competence is one of the most important dimensions underlying family functioning, which makes it important to have valid measures to assess it in different populations and in different contexts. For this reason, this study aimed to examine the psychometric properties of the Parenting Sense of Competence (PSOC; Johnston and Mash [38]), a scale that evaluates the feeling of satisfaction and the perception of efficacy in the parenting role, in both a community and an at-risk Portuguese sample.

Four different structural models (unifactorial, two-factor, three-factor, and two-factor revised) were tested, and the two-factor revised presented the best fit. This finding confirmed the original two-factor structure proposed by the PSOC authors [38] and replicated in previous studies [4,43,46,50]. It contrasts with other proposed configurations with a three-factor [49,51] or four-factor structure in a community sample [39].

Despite the replication of the original two-factor structure, it was necessary to remove six items (1, 2, 6, 8, 12, 16) since their loadings scored below the recommend value [61,62]. Analyses showed that this revised model allowed the most acceptable fit in comparison to the other tested models. An instrument structure with 10 items had already been reported in another validation study of the PSOC with mothers of Spanish at-risk families [45]. However, in this cited study, the factors were categorized as perception of effectiveness and controllability, whereas our analysis allowed us to maintain the original categorizations.

In the current study, the final structure of the PSOC is composed of two factors related to parental efficacy and satisfaction and comprises ten items, with five items per factor. Both factors (efficacy and satisfaction) retained most of the items in the original dimensions. This model presented satisfactory results regarding mean inter-item correlations and corrected item-total correlation range [65] and acceptable levels of internal consistency for both community and at-risk samples, confirming the reliability of the measure. In addition, although average variance extracted is under 0.5, composite reliability is higher than 0.6, so convergent validity of the construct can be considered adequate.

Measurement invariance of the PSOC across samples (community and at-risk parents) was also assessed, and the results of configural model in both samples presented an acceptable fit [61,63], which lends support to the measurement invariance of this scale. This indicates that the PSOC may be a suitable measure to assess PSC regardless of the context in which the family system is nested.

Furthermore, the PSOC was positively and significantly associated with parenting alliance and marital satisfaction, which was in line with our theoretical expectations. Existing literature has linked PSC with features of the dyadic relationship between mothers and fathers, such as higher parenting alliance being related with higher perceived competence [66,67]. Plus, marital satisfaction has been seen as a protective factor linked to a better sense of competence for both parents [5,68].

Additionally, the PSOC correlated negatively with parenting stress in both samples, but this association was significative only in the at-risk sample. Prior investigations have proposed that the sense of competence of at-risk parents could have distinct characteristics from community families [45,69]. In contexts of psychosocial risk, parents may have a distorted self-evaluation of their abilities to perform the parenting role, which helps them cope with the multiple demands in other domains. Thus, these parents can develop a benevolent perception of their competence as highly effective, which serves as a protective factor, reducing the stress experienced with other difficult life situations [70].

Regarding the average scores obtained in each subscale, there were no significant differences between the scores of fathers and mothers of both samples, which is in line with the study of Nunes and Ayala-Nunes [36] that also included at-risk Portuguese families. However, parents (both mothers and fathers) from the community reported more satisfaction with parenting than at-risk parents did, similarly to the finding previously reported by Seabra-Santos and colleagues [50]. This seems to reflect that community parents obtain more satisfaction than those whose contexts are, in themselves, challenging. This difference seems plausible since at-risk parents struggle with more constrains and social stress, which can preclude them to obtain higher satisfaction with the parenting role, as community parents do [69].

Taking all these findings together, this psychometric analysis allows us to recognize the PSOC as a valid and reliable instrument to evaluate PSC in both community and at-risk parents. The factorial model found in this study bring some advantages, as it is a shorter version of the scale and of easy application and interpretation, making it a useful tool for researchers and professionals. A major strength of this investigation is that both mothers and fathers where included, whereas prior studies had a sample comprised mostly of mothers. Still, fewer fathers than mothers were included. Future research should strive to have a balanced sample composition in terms of gender. The sample was comprised by parents of children with a broad age range, overcoming the limitation of previous validations of the PSOC in Portugal, which only included parents of pre-school aged children [49,50]. 

At the practical implications level, we can emphasize that the PSOC is an instrument that is easy to apply and interpret, which may be valuable not only for the empirical study of this construct but also for practitioners in intervention and family-preservation programs.

Despite the relevance of our findings, an important limitation lies in the snowball sampling technique, which precludes the guarantee of sample representativeness. Future studies should aim to replicate the exploration of the psychometric characteristics of PSOC with 10 items, using stricter sampling procedures, considering test-retest reliability, and applying an item-by-item analysis, which allows for more detailed analysis of differences in community parents and clinical populations on particular dimensions of the sense of parental competence. In addition, this study should be repeated with larger samples in order to examine small groups to assess whether, for example, parenting children at different ages or gender influences PSC.

## 5. Conclusions

The current research addressed the assessment of PSC of both community and at-risk parents using the Parenting Sense of Competence (PSOC; Johnston and Mash [38]). A revised model of the PSOC revealed appropriate validity and reliability properties. Invariance across community and at-risk samples also supported PSOC usefulness in both populations. Our conclusions highlight the suitability of this instrument as an important tool to assess Portuguese parents’ perceived satisfaction and efficacy with the parenting role.

## Figures and Tables

**Table 1:** Goodness of Fit Indices for Different ML Models of PSOC (at-risk and community sample).

PSOC	*S-Bc^2^*/*df*	IFI	NNFI	CFI	RMSEA	Confidence Interval (90%)	AIC
**At-risk sample**							
Unifactorial model	13.32	0.48	0.40	0.48	0.16	0.15–0.17	1177.22
Two-Factor model	4.04	0.87	0.85	0.87	0.08	0.07–0.09	210.38
Three-Factor model	3.34	0.92	0.91	0.92	0.07	0.06–0.08	99.41
Two-Factor model revised (1)	3.72	0.94	0.91	0.94	0.07	0.06–0.09	56.67
**Community sample**							
Unifactorial model	6.41	0.44	0.35	0.44	0.16	0.15–0.17	458.21
Two-Factor model	2.90	0.84	0.81	0.84	0.10	0.08–0.11	92.71
Three-Factor model	2.19	0.90	0.78	0.90	0.08	0.06–0.09	14.04
Two-Factor model revised (1)	1.79	0.96	0.95	0.96	0.06	0.03–0.09	−6.87

Note. ML, maximum likelihood; S-Bχ^2^, Satorra–Bentler chi-square; df, degrees of freedom; IFI incremental fit index; CFI, comparative fit index; NNF, non-normed fit index; RMSEA, root mean square error of approximation; AIC, Akaike information criterion, (1) without items 1, 2, 6, 8, 12, and 16 and intercorrelation error between items 13 and 14. Item 1: “The problems of taking care of a child are easy to solve once you know how your actions affect your child, an understanding I have acquired”; Item 2: “Even though being a parent could be rewarding, I am frustrated now while my child is at his/her present age”; Item 6: “I would make a fine model for a new mother/father to follow in order to learn what she/he would need to know in order to be a good parent”; Item 8: “A difficult problem in being a parent is not knowing whether you’re doing a good job or a bad one”; Item 12: “My talents and interests are in other areas, not in being a parent”; Item 16: “Being a parent makes me tense and anxious”.

**Table 2:** Items loadings for PSOC (at-risk sample/community sample).

PSOC		F1 (Efficacy) At-Risk Sample	F2 (Satisfaction) At-Risk Sample	F1 (Efficacy) Community Sample	F2 (Satisfaction) Community Sample
Item 3	I go to bed the same way I wake up in the morning, feeling I have not accomplished a whole lot.		0.53		0.55
Item 4	I do not know why it is, but sometimes when I’m supposed to be in control, I feel more like the one being manipulated.		0.53		0.61
Item 5	My mother/father was better prepared to be a good mother/father than I am.		0.41		0.45
Item 7	Being a parent is manageable, and any problems are easily solved.	0.30		0.43	
Item 9	Sometimes I feel like I’m not getting anything done.		0.45		0.59
Item 10	I meet my own personal expectations for expertise in caring for my child.	0.59		0.60	
Item 11	If anyone can find the answer to what is troubling my child. I am the one.	0.51		0.53	
Item 13	Considering how long I’ve been a mother/father, I feel thoroughly familiar with this role.	0.66		0.61	
Item 14	If being a mother/father of a child were only more interesting, I would be motivated to do a better job as a parent.		0.41		0.43
Item 15	I honestly believe I have all the skills necessary to be a good mother/father to mv child.	0.54		0.51	

Note. F, factor.

**Table 3:** Tests for invariance of the PSOC goodness of fit statistics.

Model	*S-Bχ*^2^ (*df*)	*∆S-Bχ*^2^ (*df*)	*CFI (ΔCFI)	*RMSEA (ΔRMSEA)
Cross sample (at-risk/community)				
1. Configural model (no constrains)	187.45(66)	-	0.94 (-)	0.07 (-)
2. Weak (metric) invariance	195.70(74)	8.23(8)*^ns^*	0.94 (.00)	0.07 (.00)
3. Strong (scalar) invariance	199.96(77)	12.42(11)*^ns^*	0.94 (.00)	0.07 (.00)

Note. S-Bχ^2^(df), Satorra–Bentler chi-square (degrees of freedom); *CFI, robust comparative fit index; *RMSEA, robust root mean square error of approximation; C.I., confidence interval; ns, non-significant.

**Table 4:** Cronbach’s alphas, mean inter-item correlations, and corrected item–total correlation range.

	Alpha	Omega	CR	AVE	MIIC	CITCR
At-risk sample						
Efficacy	0.72	0.79	0.79	0.45	0.35	0.28–0.62
Satisfaction	0.70	0.75	0.75	0.38	0.27	0.33–0.49
Community sample						
Efficacy	0.75	0.78	0.78	0.42	0.39	0.43–0.64
Satisfaction	0.76	0.77	0.77	0.41	0.38	0.39–0.62

Note. Alpha, Cronbach’s alpha; CR, composite reliability; AVE, average variance extracted; MIIC, mean inter-item correlation; CITCR, corrected item–total correlation range.

**Table 5:** PSOC criteria validity in at-risk (N = 273) and community (N = 205) sample.

	Efficacy	Satisfaction
Parenting Alliance	0.34 ***/0.25 ***	0.17 **/0.14 *
Marital Satisfaction	0.28 ***/0.24 **	0.19 **/0.41 ***
Parenting Stress Index	−0.36 ***/−0.06	−0.48 ***/−0.13

* *p* < 0.05, ** *p* < 0.01, *** *p* < 0.001.

**Table 6:** Comparisons of efficacy and satisfaction scores between at-risk (N = 273) and community (N = 205) sample.

	At-Risk *M* (*DP*)	Community *M* (*DP*)	*F*	*p*	*η^2^*
Efficacy	22.47 (4.45)	22.22 (4.04)	0.41	0.523	0.00
Satisfaction	18.35 (5.12)	21.63 (4.58)	52.62	0.000	0.10

**Table 7:** Comparisons between mothers’ (N = 309) and fathers’ (N = 144) in PSOC scores.

	Mothers *M* (*DP*)	Fathers *M* (*DP*)	*F*	*p*	*η^2^*
At-risk sample					
Efficacy	22.49 (4.45)	22.41 (4.52)	0.01	0.911	0.00
Satisfaction	18.29 (5.33)	18.63 (4.05)	0.18	0.668	0.00
Community sample					
Efficacy	22.59 (3.60)	21.82 (4.46)	1.88	0.172	0.01
Satisfaction	21.65 (4.46)	21.60 (4.73)	0.01	0.935	0.00

## Data Availability

Data can be available to consultation when request to the corresponding author.

## References

[1] Johnston C., Park J.L., Miller N.V., Sanders M.R., Morawska A. (2018). Parental cognitions: Relations to parenting and child behavior. Handbook of Parenting and Child Development Across the Lifespan.

[2] Jones T., Prinz R.J. (2005). Potential roles of parental self-efficacy in parent and child adjustment: A review. Clin. Psychol. Rev..

[3] Montigny F., Lacharité C. (2005). Perceived parental efficacy: Concepts analysis. J. Adv. Nurs..

[4] Ohan J.L., Leung D.W., Johnston C. (2000). The parenting sense of competence scale: Evidence of a stable factor structure and validity. Can. J. Behav. Sci..

[5] Sevigny P.R., Loutzenhiser L. (2010). Predictors of parenting self-efficacy in mothers and fathers of toddlers. Child Care Health Dev..

[6] Favez N., Tissot H., Frascarolo F., Stiefel F., Despland J.N. (2016). Sense of competence and beliefs about parental roles in mothers and fathers as predictors of coparenting and child engagement in mother–father–infant triadic interactions. Infant Child Dev..

[7] Van Eldik W.M., Prinzie P., Deković M., de Haan A.D. (2017). Longitudinal associations between marital stress and externalizing behavior: Does parental sense of competence mediate processes?. J. Fam. Psychol..

[8] Álvarez M., Byrne S., Rodrigo M.J. (2020). Patterns of individual change and program satisfaction in a positive parenting program for parents at psychosocial risk. Child Fam. Soc. Work.

[9] Álvarez M., Byrne S., Rodrigo M.J. (2021). Social support dimensions predict parental outcomes in a Spanish early intervention program for positive parenting. Child. Youth Serv. Rev..

[10] Baiverlin A., Gallo A., Blavier A. (2020). Impact of different kinds of child abuse on sense of parental competence in parents who were abused in childhood. Eur. J. Trauma Dissociation.

[11] Nogueira S., Abreu-Lima I., Canário C., Cruz O. (2021). Group Triple P–A randomized controlled trial with low-income mothers. Child Youth Serv. Rev..

[12] Nunes C., Ayala-Nunes L., Martins C., Gonçalves A., Nunes C., Ayala-Nunes L. (2019). As famílias em risco psicossocial no Algarve [Families at psychosocial risk in the Algarve]. Famílias em Risco. Avaliação e Intervenção Psicoeducativa [Families at Risk. Psychoeducational Assessment and Intervention].

[13] Sanders M.R., Cann W., Browne K.D., Hanks H., Stratton P., Hamilton C. (2002). Promoting positive parenting as an abuse prevention strategy. Early Prediction and Prevention of Child Abuse: A Handbook.

[14] Begle A.M., Dumas J.E. (2011). Child and parental outcomes following involvement in a preventive intervention: Efficacy of the PACE program. J. Prim. Prev..

[15] Mammen O., Kolko D., Pilkonis P. (2003). Parental Cognitions and Satisfaction: Relationship to Aggressive Parental Behavior in Child Physical Abuse. Child Maltreat..

[16] Ardelt M., Eccles J.S. (2001). Effects of mothers’ parental efficacy beliefs and promotive parenting strategies on inner-city youth. J. Fam. Issues.

[17] Farkas C., Valdés N. (2010). Maternal stress and perceptions of self-efficacy in socioeconomically disadvantaged mothers: An explicative model. Infant Behav. Dev..

[18] Albanese A.M., Russo G.R., Geller P.A. (2019). The role of parental self-efficacy in parent and child well-being: A systematic review of associated outcomes. Child Care Health Dev..

[19] Teti D.M., Candelaria M.A., Bornstein M.H. (2002). Parenting competence. Handbook of Parenting.

[20] Ayala-Nunes L., Lemos I., Nunes C. (2014). Predictores del estrés parental en madres de familias en riesgo psicosocial. Univ. Psychol..

[21] Crnic K., Ross E., Deater-Deckard K., Panneton R.K. (2017). Parenting stress and parental efficacy. Parental Stress and Early Child Development.

[22] Jackson C.B., Moreland A.D. (2018). Parental competency as a mediator in the PACE parenting program’s short and long-term effects on parenting stress. J. Child Fam. Stud..

[23] Gross D., Sambrook A., Fogg L. (1999). Behavior problems among young children in low-income urban day care centers. Res. Nurs. Health.

[24] Teti D.M., Gelfand D.M. (1991). Behavioral competence among mothers of infants in the first year: The mediational role of maternal self-efficacy. Child Dev..

[25] Porter C.L., Hsu H.C. (2003). First-time mothers’ perceptions of efficacy during the transition to motherhood: Links to infant temperament. J. Fam. Psychol..

[26] Bornstein M.H., Putnick D.L., Suwalsky J.T. (2018). Parenting cognitions→ parenting practices→ child adjustment?: The standard model. Dev. Psychopathol..

[27] Coleman P.K., Karraker K.H. (1997). Self-efficacy and parenting quality: Findings and future applications. Dev. Rev..

[28] Egberts M.R., Prinzie P., Deković M., de Haan A.D., van den Akker A.L. (2015). The prospective relationship between child personality and perceived parenting: Mediation by parental sense of competence. Pers. Individ. Differ..

[29] Gelkopf M., Jabotaro S.E. (2013). Parenting style, competence, social network and attachment in mothers with mental illness. Child Fam. Soc. Work..

[30] Shumow L., Lomax R. (2002). Parental efficacy: Predictor of parenting behavior and adolescent outcomes. Parent. Sci. Pract..

[31] Izzo C., Weiss L., Shanahan T., Rodriguez-Brown F. (2000). Parental Self-Efficacy and social support as predictors of parenting practices and children’s socioemotional adjustment in mexican immigrant families. J. Prev. Interv. Community.

[32] Hill N.E., Bush K.R. (2001). Relationships between parenting environment and children’s mental health among African American and European American mothers and children. J. Marriage Fam..

[33] Coleman P.K., Karraker K.H. (2000). Parenting self-efficacy among mothers of school-age children: Conceptualization, measurement, and correlates. Fam. Relat..

[34] Peterson G.W., Bush K.R., Gullotta T., Plant R., Evans M. (2015). Families and Adolescent Development. Handbook of Adolescent Behavioral Problems.

[35] Ayala-Nunes L., Jiménez L., Jesus S., Nunes C., Hidalgo V. (2018). An Ecological Model of Well-Being in Child Welfare Referred Children. Soc. Indic. Res..

[36] Nunes C., Ayala-Nunes L. (2017). Parenting sense of competence in at psychosocial risk families and child well-being. Bordón. Rev. Pedagog..

[37] Heerman W.J., Taylor J.L., Wallston K.A., Barkin S.L. (2017). Parenting self-efficacy, parent depression, and healthy childhood behaviors in a low-income minority population: A cross-sectional analysis. Matern. Child Health J..

[38] Johnston C., Mash E.J. (1989). A measure of parenting satisfaction and efficacy. J. Clin. Child Psychol..

[39] Gilmore L., Cuskelly M. (2008). Factor structure of the parenting sense of competence scale using a normative sample. Child Care Health Dev..

[40] Rogers H., Matthews J. (2004). The parenting sense of competence scale: Investigation of the factor structure, reliability, and validity for an Australian sample. Aust. Psychol..

[41] Pardo M.B.L., de Carvalho M.M.S.B., Primi R., Freitas D.F. (2018). Parenting Sense of Competence: A study of the psychometric properties of the scale with a Brazilian sample. Avaliação Psicol. Interam. J. Psychol. Assess.

[42] Moura D., Holanda S. (2020). Escala de Senso de Competência Parental (PSOC): Evidências de validade e precisão em contexto brasileiro [Parenting sense of competence scale (psoc): Validity and accuracy evidence in brazilian context]. Rev. Psicol. Fortaleza.

[43] Ngai F.-W., Chan S.W.-C., Holroyd E. (2007). Translation and validation of a Chinese version of the parenting sense of competence scale in Chinese mothers. Nurs. Res..

[44] Jiménez-Morago J.M., León E., Algeciras C. (2018). Parental sense of competence among non-kin foster careers from Spain. Child. Youth Serv. Rev..

[45] Menéndez S., Jiménez L., Hidalgo V. (2011). Estructura factorial de la escala PSOC (parental sense of competence) en una muestra de madres usuarias de servicios de preservación familiar [Factorial structure of PSOC (Parental Sense of Competence) scale with a sample of mothers from family preservation services]. Rev. Iberoam. Diagn. Eval. Psicol..

[46] Oltra-Benavent P., Cano-Climent A., Oliver-Roig A., Cabrero-García J., Richart-Martínez M. (2020). Spanish version of the Parenting Sense of Competence scale: Evidence of reliability and validity. Child Fam. Soc. Work.

[47] Suwansujarid T., Vatanasomboon P., Gaylord N., Lapvongwatana P. (2013). Validation of the parenting sense of competence scale in fathers: Thai version. Southeast Asian J. Trop. Med. Public Health.

[48] Augustinavicius J.L., Murray S.M., Familiar-Lopez I., Boivin M.J., Mutebe A., Arima E., Bass J.K. (2020). Measurement of parenting self-efficacy among female hiv-affected caregivers in Uganda. Matern. Child Health J..

[49] Ferreira B., Monteiro L., Fernandes C., Cardoso J., Veríssimo M., Santos A.J. (2014). Percepção de competência parental: Exploração de domínio geral de competência e domínios específicos de auto-eficácia, numa amostra de pais e mães portuguesas [Perception of parental competence: Exploration of the general domain of competence and specific domains of self-efficacy, in a sample of Portuguese fathers and mothers]. Anal. Psicol..

[50] Seabra-Santos M.J., Major S., Pimentel M., Gaspar M.F., Antunes N., Roque V. (2015). Escala de Sentido de Competência Parental (PSOC): Estudos psicométricos [Parenting Sense of Competence Scale (PSOC): Psychometric studies.]. Avaliação Psicol. Interam. J. Psychol. Assess..

[51] Nunes C., Jiménez L., Menéndez S., Ayala-Nunes L., Hidalgo V. (2016). Psychometric properties of an adapted version of the parental sense of competence (PSOC) scale for Portuguese at-risk parents. Child Fam. Soc. Work.

[52] Tabachnick B.G., Fidell L.S. (2019). Using Multivariate Statistics.

[53] Byrne S., Rodrigo M.J., Martín J.C. (2012). Influence of form and timing of social support on parental outcomes of a child-maltreatment prevention program. Child. Youth Serv. Rev..

[54] Abidin R.R., Brunner J.F. (1995). Development of a parenting alliance inventory. J. Clin. Child Psychol..

[55] Nunes C., Ayala-Nunes L., Martins C., Pechorro P., Ferreira L.I. (2021). Parenting Alliance Inventory: Psychometric properties and invariance among a community and at-risk sample of Portuguese parents. J. Child Fam. Stud..

[56] Fowers B.J., Olson D.H. (1993). ENRICH Marital Satisfaction Scale: A brief research and clinical tool. J. Fam. Psychol..

[57] Nunes C., Ferreira L.I., Martins C., Pechorro P., Ayala-Nunes L. (2022). The enrich marital satisfaction scale: Adaptation and psychometric properties among at-risk and community Portuguese parents. J. Soc. Pers. Relatsh..

[58] Abidin R.R. (1995). Parenting Stress Index (PSI). Manual.

[59] Santos S. Forma reduzida do Parenting Stress Index (PSI): Estudo preliminar [Short form of the Parenting Stress Index (PSI): Preliminary study, Paper presentation]. Proceedings of the XIII Conferência Internacional Avaliação Formas e Contextos.

[60] Bentler P.M., Wu E.J. (2015). Supplement to EQS 6.3 for Windows User’s Guide.

[61] Sahoo M., Subudhi R.N., Mishra S. (2019). Structural Equation Modeling: Threshold Criteria for Assessing Model Fit. Methodological Issues in Management Research: Advances, Challenges, and the Way Ahead.

[62] Marôco J. (2021). Análise de Equações Estruturais [Structural Equation Analysis].

[63] Byrne B.M., van de Vijver F.J. (2017). The maximum likelihood alignment approach to testing for approximate measurement invariance: A paradigmatic cross-cultural application. Psicothema.

[64] Cheung G.W., Rensvold R.B. (2002). Evaluating goodness-of-fit indexes for testing measurement invariance. Struct. Equ. Model..

[65] Nunnally J., Bernstein I. (1994). Psychometric Theory.

[66] Flouri E. (2005). Fathering and Child Outcomes.

[67] Floyd F.J., Gilliom L.A., Costigan C.L. (1998). Marriage and the parenting alliance: Longitudinal prediction of change in parenting perceptions and behaviors. Child Dev..

[68] Belsky J., Youngblade L., Rovine M., Volling B. (1991). Patterns of marital change and parent-child interaction. J. Marriage Fam..

[69] Rodrigo M.J., Martín J.C., Máiquez M.L., Rodríguez G. (2007). Informal and formal supports and maternal child-rearing practices in at-risk and non at-risk psychosocial contexts. Child. Youth Serv. Rev..

[70] Rodrigo M.J., Byrne S. (2011). Social support and personal agency in at-risk mothers. Psychosoc. Interv..

